# Does Expertise Reduce Rates of Inattentional Blindness? A Meta-Analysis

**DOI:** 10.1177/03010066211072466

**Published:** 2022-01-21

**Authors:** Malin Ekelund, Hanna Fernsund, Simon Karlsson, Erik Mac Giolla

**Affiliations:** 3570 University of Gothenburg, Sweden

**Keywords:** attention, inattention/attention blindness, expertise, meta-analysis

## Abstract

Inattentional blindness occurs when one fails to notice a fully visible stimulus because one's attention is on another task. Researchers have suggested that expertise at this other task should reduce rates of inattentional blindness. However, research on the topic has produced mixed findings. To gain clarity on the issue, we meta-analyzed the extant studies (*K* = 14; *N* = 1153). On average, experts showed only a slight reduction in rates of inattentional blindness: 62% of novices experienced inattentional blindness compared to 56% of experts, weighted odds ratio = 1.33, 95% CI [0.78, 2.28]. The relevance of the stimuli to the experts’ domain of expertise showed no notable moderating effects. The low number of the included studies, and the small sample sizes of the original studies, weaken our conclusions. Nonetheless, when taken together, the available evidence provides little support for any reliable influence of expertise on rates of inattentional blindness.

Inattentional blindness is the phenomenon of failing to detect fully visible stimuli present in one's visual field when attention is focused on something else (Mack & Rock, 1998; Neisser & Becklen, 1975; for a review see Jensen et al., 2011). It is a well-documented phenomenon that has been shown to occur in a wide variety of lab-based (e.g., Most et al., 2001; Pammer et al., 2018; Simons & Chabris, 1999) and real-world settings (e.g., Chabris et al., 2011; Hyman et al., 2009; Simons, 2010; Simons & Schlosser, 2017). Similarly, it has been demonstrated using a broad range of stimuli, from simple shapes (Most et al., 2005) to a simulated real-world assault (Chabris et al., 2011). Studies on inattentional blindness follow the same basic procedure: participants take part in an attention-demanding primary task, during which an unexpected visual stimulus is presented in the participant's visual field. Participants then report whether or not they noticed the unexpected stimulus. Participants who do not notice the stimulus have experienced inattentional blindness.

The phenomenon is strikingly demonstrated in Simons and Chabris’ (1999) classic study. In the study, participants tasked with counting the number of basketball passes made by a group of players, regularly failed to report the highly visible unexpected stimulus—someone dressed as a gorilla—despite it appearing directly in their visual field. Moving beyond simply demonstrating the effect, much contemporary research attempts to define under what conditions inattentional blindness occurs, and which factors may influence rates of experiencing inattentional blindness. Here, we focus on one of these proposed factors, namely, expertise.

Expertise—a high level of knowledge or skill in a specific domain (Ericsson, 2006)—has been proposed as an important moderator of inattentional blindness. Most argue that expertise at the primary task should reduce the risk of inattentional blindness. This is because expertise should facilitate visual information processing, reducing the attentional resources occupied by the primary task (Drew et al., 2013; Simons & Schlosser, 2017), and allow for faster and more efficient processing of the search space (Pammer et al., 2018). Contrary to this viewpoint, others have suggested that expertise may in fact increase the risk of inattentional blindness. This is because experts may be more conditioned to focus on the primary task, or, alternatively, that younger non-experts are simply more alert, focused, attentive, and better at multitasking (Ho et al., 2017).

These conflicting theoretical accounts are reflected in the inconsistent empirical findings. Some studies show that expertise is associated with a reduction in inattentional blindness (e.g., Drew et al., 2013; Memmert, 2006; Simons & Schlosser, 2017). For example, Simons and Schlosser (2017) found that experienced police officers, compared to police trainees, were more likely to notice an unexpected gun during a simulated vehicle traffic stop. Other studies have found there to be a weak association between expertise and inattentional blindness (e.g., Näsholm et al., 2014). For example, Näsholm et al., found that infantry personnel, with considerable experience in monitoring CCTV footage, were no more likely than members of the public to notice a woman acting suspiciously in the background of a video they were asked to monitor. Finally, some studies have even found that expertise is associated with an increase in inattentional blindness. For example, Ho et al. (2017) found that medical students were more proficient than experienced anesthesiologists at detecting unusual head movements during a simulated abdominal surgery.

One explanation for these disparate findings is that the proposed moderating effect of expertise on inattentional blindness is influenced by other moderators. From the literature, we identified one moderator of particular theoretical interest: the relevance of the unexpected stimulus to the expert's domain of expertise.

Previous research suggests that both the familiarity and meaningfulness of the unattended stimulus can affect rates of inattentional blindness—with more familiar or meaningful stimuli being more regularly noticed (Mack & Rock, 1998). For example, Mack and Rock found that more familiar words, such as ‘the’ and ‘and’, were more regularly noticed than less familiar words, such as ‘tie’ and ‘ant’. Similarly, they found that more meaningful stimuli, such as a schematic depiction of a smiling face, are more regularly noticed than less meaningful stimuli, such as similarly sized scrambled faces or circles.

Applied to the current context, the moderating roll of familiarity or meaningfulness on rates of inattentional blindness may interact with expertise. Specifically, if the unattended stimulus bears particular relevance to the expert's domain, experts may find the stimulus more familiar or meaningful compared to novices, and hence show lower rates of inattentional blindness (Pammer et al., 2018). Conversely, any benefit of expertise may be reduced if the unexpected stimulus is of little relevance to the expert's domain of expertise.

Studies on expertise and inattentional blindness have used unexpected stimuli that have higher and lower levels of relevance to the expert's domain. An example of an unexpected stimulus with high domain relevance is the presence of a gun during a vehicle stop-and-search (Simons & Schlosser, 2017). It is plausible, for instance, that police officers’ experiences makes them more familiar with guns compared to laypeople, and hence more attuned to noticing them. In contrast, an image of a gorilla in computed tomography (CT) scans of a lung (Drew et al., 2013) is an example of an unexpected stimulus with low domain relevance, since images of gorillas have no particular relevance to radiologists.

Here, we conduct a meta-analysis on studies examining the association between expertise and inattentional blindness. In addition, we examine the moderating effect of the relevance of the unexpected stimulus to the experts’ domain. In doing so, we hope to gain a greater understanding of the inconsistent findings in the field.

## Methods

### Inclusion Criteria Characteristics of the Literature

For studies to be included in this meta-analysis, they had to meet three inclusion criteria. First, they should have explicitly examined inattentional blindness. Second, they should have compared rates of inattentional blindness among people with more expertise in a specific domain to people with less expertise in the same domain. For ease, we refer to those with more expertise as ‘experts’ and those with less expertise as ‘novices’, while acknowledging that these categorizations are fraught with definitional issues (Ericsson, 2006). Third, participants had to be over 18 years old. This was included so as not to confound age and expertise.

### Search Strategy

**Figure 1.**
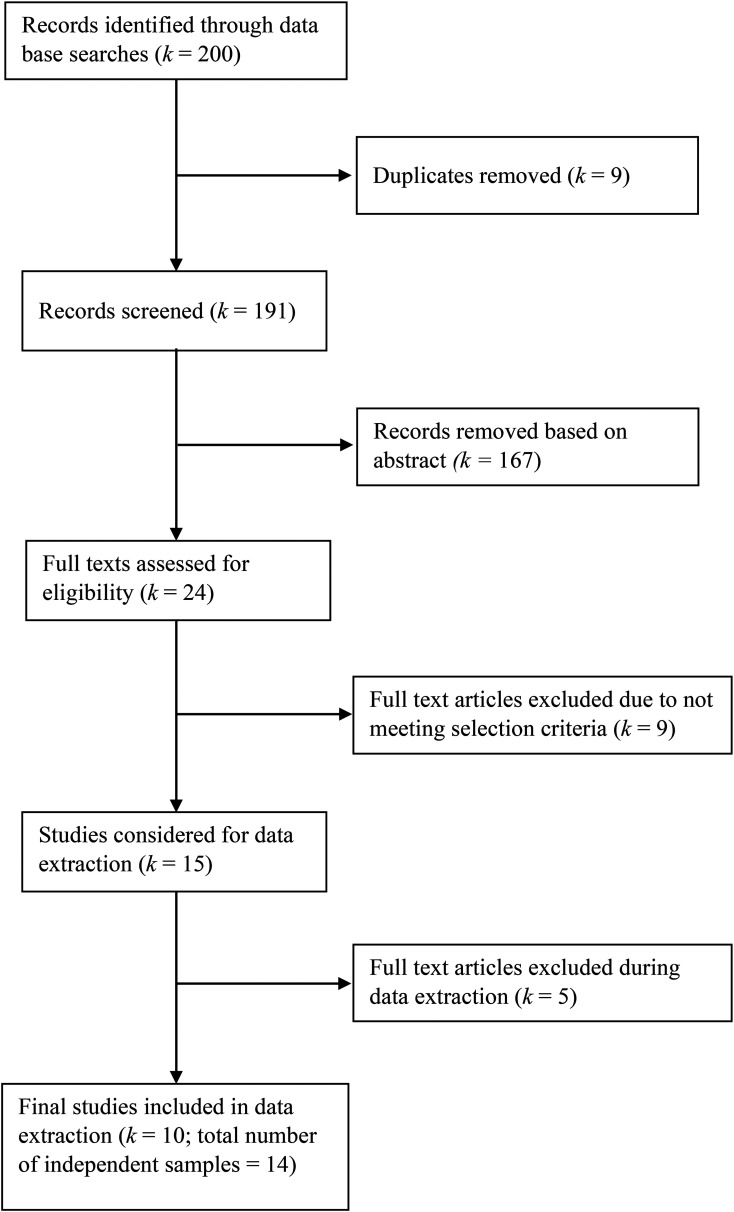
Overview of screening procedure.

A literature search was conducted in April 2020. We used the database PsycINFO and the search engine Google Scholar to search for empirical studies. In PsycINFO, we used the search string ‘inattentional blindness’ AND expert* OR experience*. In Google Scholar, we used the search string *inattentional blindness AND expert*. Due to the high number of hits on Google Scholar, we limited our search to the first 100 hits. To search for additional grey literature, we also searched the database ProQuest Dissertations & Theses Global using the search string ‘inattentional blindness’ AND expert*, as well as Open Access Theses and Dissertations using the search string *inattentional blindness AND expert*. To organize the search results from these four databases and search engines, we used the web-based application Rayyan (rayyan.qcri.org). Excluding duplicates, the search yielded 191 hits. Out of these, 13 studies met the inclusion criteria, resulting in 17 independent samples (see Figure 1 for a PRISMA flowchart of the exclusion process).

In addition to this, we also searched for additional studies that met the inclusion criteria by searching through reference lists of relevant articles and author bibliographies. This additional search strategy did not yield any further studies to be included. Finally, we contacted the first authors of the 13 studies to request unpublished ‘file-drawer’ studies. None of the authors had any unpublished studies, with the exception of a recently submitted article, which we were unable to include due to its status as under review.

During the journal's review process, we subsequently removed a further three studies: Cozza (2010); Memmert et al. (2009), and Pammer and Blink (2018). Although these studies compared experts to novices on an inattentional blindness task, and hence fulfilled our inclusion criteria, the primary tasks were deemed too removed from the experts’ domain of expertise. Cozza compared people who had completed a mindfulness-based stress reduction class to a control condition; Memmert et al. compared athlete handball players to novice athletes; while Pammer and Blink compared federal police with fast pursuit driving training to novice drivers. However, in all of these studies, the primary tasks consisted of monitoring simple shapes on a computer screen. We argue that the link between expertise and the primary task in these studies is too indirect to be a genuine test of the relationship between expertise and inattentional blindness.

### Characteristics of the Literature

In total, we found 14 samples fitting our inclusion criteria. The 14 samples came from 10 articles issued in peer-reviewed journals, published between 2006 and 2018. The 14 samples included 1153 individuals. Of these, the original authors categorized 526 as experts and 627 as novices. For an overview of all the samples, see Table 1.

**Table 1.** Overview of studies examining expertise and inattentional blindness.

Sample	*N* experts	Description of experts	*N* novices	Description of novices	Primary task	Unexpected stimuli	Domain relevant
Al-Moteri et al. (2018)	N = 21 Hits = 10 Misses = 11	Nurses with two or more years of experience in a hospital ward.	N = 19 Hits = 16 Misses = 3	Nurses with less than 2 years of experience in a hospital ward.	An 8 min screen-based interactive simulation of a patient suffering from hypovolemic shock who is acutely deteriorating. Participants diagnosed and managed the patient by collecting vital cues by interacting with the simulation with mouse clicks.	Clinically relevant diagnostic cues.	Yes
Drew et al. (2013)	N = 24 Hits = 4 Misses = 20	Radiologists.	N = 25 Hits = 0 Misses = 25	Naïve observers with no prior medical training.	Participants scrolled through five stacks of CT scans searching for lung nodules. Each trial contained on average 10 nodules. Participants had approximately 3 min to freely scroll through each trial.	In the final trial (which contained 239) slices, an image of a gorilla appeared on five slices at varying levels of opacity. The gorilla measured 29 × 50 mm.	No
Furley et al. (2010)	N = 13 Hits = 9 Misses = 4	Competitive basketball players who had continuously been playing in the fourth highest league in Europe, or higher.	N = 12 Hits = 5 Misses = 7	Students who had taken a basic basketball class, but had never played basketball competitively.	Video sequences (approximately 15 s each) of a five-on-five basketball game situation involving two adult teams, in which five attackers wearing green basketball jerseys were playing five defenders wearing white jerseys. At the end of the video, one of the attacking players received the ball. Participants were asked to take over the role of this player and to make a tactical decision for this player by saying out loud their next move, for example, ‘pass to left wing player’, ‘cut’, or ‘shoot’.	In the 4th trial, an obviously unguarded player who would be the best player to pass to if the participant perceived him, appeared closest to the defending player, on whom the participants had to focus their attention.	Yes
Greig et al. (2014)	N = 43 Hits = 13 Misses = 30	Accredited advanced life support (ALS) providers.	N = 56 Hits = 11 Misses = 45	Basic life support (BLS) providers or with no formal resuscitation training.	Participants were shown a 50 s video depicting a simulated adult resuscitation in progress. Participants were instructed to observe the team and to be prepared to comment on the appropriateness of CPR and defibrillation technique.	The oxygen supply becomes disconnected from the wall.	Yes
Ho et al. (2017)	N = 31 Hits = 10 Misses = 21	Certified anesthesiologists.	N = 46 Hits = 28 Misses = 18	Upper-year medical students.	Participants watched a video of a simulated surgery and scored the abnormalities they saw.	Patient head movements and a leaky central catheter.	Yes
Memmert (2006)	N = 24 Hits = 15 Misses = 9	Basketball players with around 12 years of experience.	N = 24 Hits = 8 Misses = 16	Undergraduate juniors.	Watch the video by Simons and Chabris (1999) where people in black or white t-shirts passed a ball to each other. The task was to count number of passes by the white team.	A person in a Gorilla costume who walks through the screen.	No
Näsholm et al. (2014 Subsample a)	N = 43 Hits = 17 Misses = 26	Infantry personnel who regularly monitored CCTV footage in their work.	N = 44 Hits = 22 Misses = 22	Members of the public or students with no experience of monitoring CCTV footage.	Participants viewed a video of a staged crime depicting a bicycle theft and a drug deal. They then filled in a questionnaire about what they had seen.	A woman walked halfway across the back of the scene, placed a suspicious parcel on the ground, stood up, looked straight into the camera, and walked off.	Yes
Näsholm et al. (2014 Subsample b)	N = 41 Hits = 10 Misses = 31	Infantry personnel who regularly monitored CCTV footage in their work.	N = 43 Hits = 8 Misses = 35	Members of the public or students with no experience of monitoring CCTV footage.	Participants viewed a video of a staged crime depicting a bicycle theft and a drug deal. They then filled in a questionnaire about what they had seen.	A woman appeared in the back of the scene wearing a pirate's costume. The pirate entered the scene, looked straight into the camera and exited.	No
Pammer et al. (2018 Subsample a)	N = 32 Hits = 0 Misses = 32	Paramedics who had completed paramedic officer driver training.	N = 37 Hits = 7 Misses = 30	Members of the public with driver's licenses, but with no special driving training.	Participants viewed a sequence of high-resolution static images of relatively normal driving scenes taken from the driver's perspective. Participants were asked to indicate whether each scene depicted a safe or unsafe driving situation from the perspective of the driver.	Garbage bin.	No
Pammer et al. (2018 Subsample b)	N = 65 Hits = 36 Misses = 29	Paramedics who had completed paramedic officer driver training.	N = 95 Hits = 40 Misses = 55	Members of the public with driver's licenses, but with no special driving training.	Participants viewed a sequence of high-resolution static images of relatively normal driving scenes taken from the driver's perspective. Participants were asked to indicate whether each scene depicted a safe or unsafe driving situation from the perspective of the driver.	Pedestrians (adult or child standing).	Yes
Pammer et al. (2018 Subsample c)	N = 25 Hits = 17 Misses = 8	Paramedics who had completed paramedic officer driver training.	N = 21 Hits = 14 Misses = 7	Members of the public with driver's licenses, but with no special driving training.	Participants viewed a sequence of high-resolution static images of relatively normal driving scenes taken from the driver's perspective. Participants were asked to indicate whether each scene depicted a safe or unsafe driving situation from the perspective of the driver.	Stroller.	Yes
Pammer et al. (2018 Subsample d)	N = 29 Hits = 28 Misses = 1	Paramedics who had completed paramedic officer driver training.	N = 36 Hits = 31 Misses = 5	Members of the public with driver's licenses, but with no special driving training.	Participants viewed a sequence of high-resolution static images of relatively normal driving scenes taken from the driver's perspective. Participants were asked to indicate whether each scene depicted a safe or unsafe driving situation from the perspective of the driver.	Child running.	Yes
Sannes et al. (2018)	N = 60 Hits = 10 Misses = 50	4th-year chiropractic students.	N = 69 Hits = 4 Misses = 65	2nd-year chiropractic students.	Participants examined 20 AP Pelvic radiographs. Participants viewed each radiograph for 30 s, and were asked to report in a questionnaire whether or not there were any findings. The questionnaire contained one such question per image.	A 29 × 50 mm image of a gorilla at varying levels of opacity appeared on 3 of the radiographs.	No
Simons and Schlosser (2017)	N = 75 Hits = 50 Misses = 25	Experienced police officers, with an average of 12 years patrol experience.	N = 100 Hits = 42 Misses = 58	Police trainees who were in the fifth or sixth week of their police academy training, had received classroom instruction on vehicle stops, and had participated in 4–8 h of vehicle stop scenarios.	Simulated (but real-world) vehicle stop. Participants were to use their discretion to decide whether to issue a traffic citation or a warning citation.	An unloaded pistol on the dashboard above the glovebox.	Yes

*Note*. Hits refer to the number of participants that noticed the unexpected stimuli. Misses refer to the number of participants that did not notice the unexpected stimuli, that is, who experienced inattentional blindness. For Ho et al. (2017) we took the average hit rate across the two unexpected stimuli. For Näsholm et al. (2014a, 2014b) we collapsed the video length conditions. For Sannes et al. (2018) we collapsed across the density conditions and used the estimates that excluded participants who were aware of the study by Drew et al. (2013). For Pammer et al. (2018a, 2018b, 2018c, 2018d), we used the online application https://apps.automeris.io/wpd/ to extract exact percentages from Figure 2. Domain relevant refers to the relevance of the unexpected stimulus to the experts’ domain of expertise.

### Analysis Plan

#### Primary Analysis

The analyses were conducted using the Metafor package (Viechtbauer, 2010) for R (R Core Team, 2019). Analyses were conducted on the log odds as this makes the outcome measure closer to normal and null effects symmetric around zero, desirable qualities for meta-analyses (Haddock et al., 1998). We opted for a random effects model as we expected substantial between-study variance due to differences in populations, primary tasks, and unexpected stimuli (Cooper, 2010). All data and code to perform the analyses are available at https://osf.io/9jzbn/?view_only=fa6bb9685ee440e3852559ee84a2c34c.

We used different approaches to investigate small study effects and potential indicators of publication bias. We investigated funnel plot asymmetry by a visual inspection of the funnel plot as well as through an Egger's test (Egger et al., 1997). If asymmetry was suspected, we used the trim and fill procedure to correct for the bias (Duval & Tweedie, 2000). We also used a selection model approach with one cutoff (.05). When using one cutoff, this form of selection model is also known as a three-parameter selection model (McShane et al., 2016).

#### Moderation Analysis

The moderator ‘domain relevance’ was dichotomously coded: high domain relevance versus low domain relevance. For a study, or a study's subsample, to be categorized as having high domain relevance the unexpected stimulus had to be considered as having a particular meaning or higher level of familiarity for experts compared to novices. For example, a head movement during an abdominal surgery is an event which is rare, but which could realistically occur, and may have been witnessed more times by experienced anesthesiologists compared to medical students (Ho et al., 2017). This would therefore be coded as having high domain relevance. In contrast, outside of a research lab, an image of a gorilla could not appear in a CT scan of a lung (Drew et al., 2013) and would not have been witnessed by even the most experienced radiologists. This would therefore be coded as having low domain relevance. Two of the authors independently coded the unexpected stimuli of the 14 samples. Coders showed 93% agreement, 13 of 14 samples. The disagreement concerned Pammer et al. (2018 Subsample a). Specifically, the study compares paramedics with special driving training to members of the public with no such training. The primary task was to judge whether images of driving scenes were safe or not. The unexpected stimulus was a garbage bin. The disagreement was resolved through a discussion between the coders, in which it was decided that the domain relevance of the stimulus should be categorized as low, since garbage bins are a minimal safety risk. For comparison, in Pammer et al. (2018 Subsample b), the unexpected stimulus was a pedestrian, a higher safety risk. This was therefore characterized as having high domain relevance. Nine of the samples were coded as having high domain relevance and five samples were coded as having low domain relevance (see Table 1).

## Results

### Main Analyses

**Figure 2.**
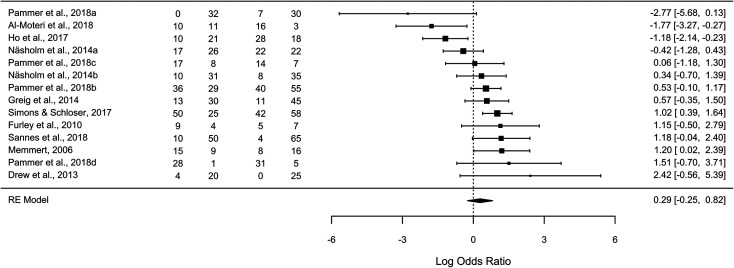
The forest plot depicts the log odds. Positive values indicate higher hit rates for experts compared to novices. Error bars depict the 95% confidence intervals. The diamond shows the meta-analytic average log odds. The width of the diamond depicts the 95% confidence interval. ‘Hits’ and ‘Misses’ are the sample level rates of hits (number who reported seeing the unexpected stimuli) and misses (number who did not report seeing the unexpected stimuli) for experts and novices, respectively.

Sixty-two percent of the novices experienced inattentional blindness, compared to 56% of experts. Figure 2 provides the rates of inattentional blindness for experts and novices for each study, as well as an overview of the impact of expertise on inattentional blindness expressed in log odds. The weighted meta-analytic average log odds was 0.29, 95% CI [−0.25, 0.82], *p* = .295, τ = .783, *I*^2^ = 67.25%. To make the results more intuitive and interpretable, this effect can be transformed to an odds ratio (OR) = 1.33, 95% CI [0.78, 2.28], 95% prediction interval (PI) [0.26, 6.76]. This estimate indicates that it is 1.33 times more likely that experts notice the unexpected stimulus compared to novices, suggesting, at best, a small overall advantage for experts.

**Figure 3.**
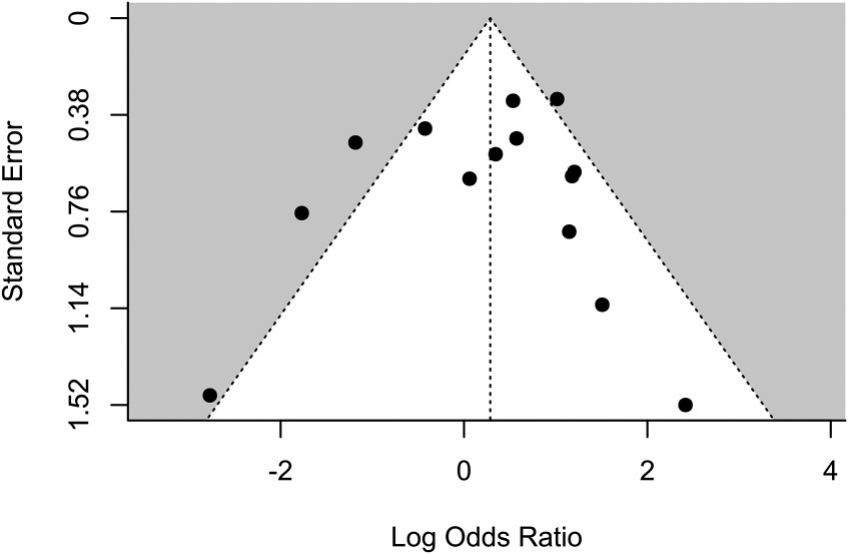
Funnel plot of the log odds of the 14 samples. Vertical line represents the meta-analytic average log odds. Dashed diagonal lines represent the 95% confidence interval for the meta-analytic estimate. Positive values indicate higher hit rates (i.e., lower levels of inattentional blindness) for experts compared to novices.

The Egger's test did not suggest any presence of publication bias (*p* = .890). However, a visual inspection of the funnel plot showed, perhaps, a slight overrepresentation of studies on the right side of the plot (see Figure 3). For this reason, we calculated bias-corrected estimates using the trim and fill procedure. The trim and fill procedure imputed one study on the left hand side of the funnel plot, producing a smaller effect than the original meta-analysis, log odds = 0.23, 95% CI [−0.31, 0.77], OR = 1.26, 95% CI [0.74, 2.15], 95% PI [0.24, 6.58], *p* = .40.

In addition, we corrected for potential bias using a selection model with one specified cutpoint (.05). This resulted in a near elimination of any trend observed in the primary analysis, log odds = 0.06, 95% CI [−0.58, 0.70], OR = 1.06, 95% CI [0.27, 4.09], 95% PI [0.24, 6.58], *p* = .86. However, precision in the estimation was also reduced, demonstrated by the wider confidence intervals. Furthermore, the reduction in effect size compared to the original analysis was not significant, indicated by the Likelihood Ratio test, *p* = .390. It should be noted that the non-significant Likelihood Ratio test could be a result of low power due to the low number of studies.

In sum, the average association between expertise and inattentional blindness observed in the published literature is small and non-significant. However, there was considerable heterogeneity in effect sizes between studies, as indicated by the wide PIs. This suggests the presence of between-study moderators.

### Moderation Analyses

Our moderation analysis compared studies with high domain relevance (*k* = 9) to studies with low domain relevance (*k* = 5). The low number of studies means the moderation analysis only has the power to reveal large moderation effects. The domain-relevance moderator was not statistically significant, *Q*(1) = 0.88, *p* = 0.348, and accounted for only 2.22% of the heterogeneity. In fact, against predictions, there was a trend in the opposite direction. In the high domain-relevance condition, experts did not show significantly lower rates of inattentional blindness compared to novices, log odds = 0.12, OR = 1.13, 95% CI [0.59, 2.16], 95% PI [0.21, 6.19], *p* = 0.705. In contrast, in the low domain-relevance condition experts did show significantly lower rates of inattentional blindness compared to novices, log odds = 0.74, OR = 2.10, 95% CI [1.01.72; 4.35], 95% PI [0.77., 5.76], *p* = 0.046. However, we strongly advise against overinterpreting this result. The primary reason being the imprecise parameter estimates due to (i) the low number of studies included in the analyses and (ii) the small sample sizes of the original studies.

## Discussion

We meta-analyzed studies examining the relationship between expertise and inattentional blindness. Overall, experts and novices differed little in rates of inattentional blindness. The relevance of the unexpected stimulus to the experts’ domain did not show any notable moderating effects. Nonetheless, the wide PIs suggest that in some circumstances expertise may have a considerable influence on rates of inattentional blindness. This suggests that other, unidentified, moderators may be at play.

Overall, however, our results provide little support for the hypothesis that expertise should reliably reduce rates of inattentional blindness. To reiterate, this hypothesis rests on two premises. First, a reduction in cognitive load during the primary task should free up attentional resources, thereby increasing one's capacity to notice unexpected stimuli. Second, expertise at the primary task should lead to such a reduction in cognitive load. There is ample evidence for the first premise—that lower cognitive load leads to lower rates of inattentional blindness (e.g., Cartwright-Finch & Lavie, 2007; Greene et al., 2017; Murphy & Greene, 2016). We therefore reason that the negligible link between expertise and inattentional blindness is due to shortcomings with the second premise.

Expertise should of course lead to improved performance on the primary task. However, this improved performance does not necessarily imply a reduction in cognitive load. Such a reduction should only occur if some degree of automaticity has been achieved on the primary task (Haith & Krakauer, 2018; Pashler, 1994). If the primary task remains sufficiently difficult for experts, a reduction in cognitive load may not be observed, even if experts perform better at the task. If no reduction in cognitive load occurs, a gain in attentional resources is unlikely. Consequently, lower rates of inattentional blindness for experts, should, perhaps, not be expected. This reasoning may also explain the related finding that individual differences in the ability to perform the primary task do not predict rates of inattentional blindness (Simons & Jensen, 2009).

As a concrete example consider the study by Drew et al. (2013), where the primary task was to detect cancer nodules in CT scans. Although experts performed better than novices on this task, experts still only detected an average of 55% of all the cancer nodules. This suggests that the primary task was difficult even for experts and may not have obtained the level of automaticity necessary to reduce cognitive load. Future research on the topic could examine whether our reasoning holds by choosing primary tasks that experts, but not novices, have developed automaticity in. At a minimum, researchers may want to choose expert and novice groups with substantial differences in domain expertise. Arguably, this has not always been the case (e.g., Sannes et al., 2018, compared 4th- to 2nd-year chiropractic students).

The low number of studies included in this meta-analysis limits the conclusions we can draw. This is compounded by the small sample sizes of the original studies. With that said, based on the available literature, our results show that, on average, expertise will have only a small effect on inattentional blindness. To detect such effects with any consistency will require considerably larger sample sizes than are currently used. For instance, to detect the observed effect in our meta-analysis—an odds ratio of 1.33—at a conventional significance level of .05, with 80% power, would require over 1000 participants—over 500 experts and 500 novices (see supplemental material S1 Figure 1). For comparison, the median sample size of studies in our meta-analysis was 73. Researchers who continue to work on the topic must take this into consideration.

Although the relevance of the unexpected stimulus to the experts’ domain did not show a clear moderating effect, our results suggest there may be more influential moderators yet to be uncovered. Specifically, the wide PIs around the average effect size suggest that under some circumstances, or with some populations, expertise can be expected to show a stronger relationship with inattentional blindness. Searching for these moderating effects may be a viable direction for future research.

Inattentional blindness is a well-established phenomenon in cognitive psychology. Researchers have rightly moved on from simply demonstrating the phenomenon to examining its moderators—asking when, where, and to whom inattentional blindness is more likely to occur. Here, we examined one such moderator: Expertise. The strength of our conclusions is limited by the small number of studies included in the meta-analysis and by limitations with the original studies. Foremost among these are the small samples typically used. Nonetheless, the collective evidence from the reviewed studies provides little support for a reliable influence of expertise on rates of inattentional blindness.

## Authors’ Note

All data and code to reproduce the analyses are available at https://osf.io/9jzbn/?view_only=fa6bb9685ee440e3852559ee84a2c34c

## Supplemental Material

sj-pdf-1-pec-10.1177_03010066211072466 - Supplemental material for Does Expertise Reduce Rates of Inattentional Blindness? A Meta-Analysis Click here for additional data file. Supplemental material, sj-pdf-1-pec-10.1177_03010066211072466 for Does Expertise Reduce Rates of Inattentional Blindness? A Meta-Analysis by Malin Ekelund, Hanna Fernsund, Simon Karlsson and Erik Mac Giolla in Perception

## References

[bibr1-03010066211072466] References marked with an asterisk were included in the meta-analysis.

[bibr2-03010066211072466] * Al-Moteri M. O. Symmons M. Cooper S. Plummer V. (2018). Inattentional blindness and pattern-matching failure: The case of failure to recognize clinical cues. Applied Ergonomics, 73, 174–182. 10.1016/j.apergo.2018.07.001 30098633

[bibr3-03010066211072466] Cartwright-Finch U. Lavie N. (2007). The role of perceptual load in inattentional blindness. Cognition, 102(3), 321–340. 10.1016/j.cognition.2006.01.002 16480973

[bibr4-03010066211072466] Chabris C. F. Weinberger A. Fontaine M. Simons D. J. (2011). You do not talk about Fight Club if you do not notice Fight Club: Inattentional blindness for a simulated real-world assault. i-Perception, 2(2), 150–153. 10.1068/i0436 23145232 PMC3485775

[bibr5-03010066211072466] Cooper H. (2010). Research synthesis and meta-analysis: A step-by-step approach (4th ed.). Sage Publications, Inc.

[bibr6-03010066211072466] Cozza C. M. (2010). *Does mindfulness affect subsystems of attention?* [Doctoral dissertation]. Duke University, DukeSpace. https://dukespace.lib.duke.edu/dspace/handle/10161/5627

[bibr7-03010066211072466] * Drew T. Võ M. L. H. Wolfe J. M. (2013). The invisible gorilla strikes again: Sustained inattentional blindness in expert observers. Psychological Science, 24(9), 1848–1853. 10.1177/0956797613479386 23863753 PMC3964612

[bibr8-03010066211072466] Duval S. Tweedie R. (2000). Trim and fill: A simple funnel-plot–based method of testing and adjusting for publication bias in meta-analysis. Biometrics, 56(2), 455–463. 10.1111/j.0006-341X.2000.00455.x 10877304

[bibr9-03010066211072466] Egger M. Smith G. D. Schneider M. Minder C. (1997). Bias in meta-analysis detected by a simple, graphical test. British Medical Journal, 315(7109), 629–634. 10.1136/bmj.315.7109.629 9310563 PMC2127453

[bibr10-03010066211072466] Ericsson K. A. (2006). An introduction to Cambridge handbook of expertise and expert performance: Its development, organization, and content. In Ericsson K. A. Charness N. Feltovich P. J. Hoffman R. R. (Eds.), Cambridge handbook of expertise and expert performance (pp. 3–20). Cambridge University Press

[bibr11-03010066211072466] * Furley P. Memmert D. Heller C. (2010). The dark side of visual awareness in sport: Inattentional blindness in a real-world basketball task. Attention, Perception, & Psychophysics, 72(5), 1327–1337. Cambridge University Press 10.3758/APP.72.5.1327 20601714

[bibr12-03010066211072466] Greene C. M. Murphy G. Januszewski J. (2017). Under high perceptual load, observers look but do not see. Applied Cognitive Psychology, 31(4), 431–437. 10.1002/acp.3335

[bibr13-03010066211072466] * Greig P. R. Higham H. Nobre A. C. (2014). Failure to perceive clinical events: An under-recognized source of error. Resuscitation, 85(7), 952–956. 10.1016/j.resuscitation.2014.03.316 24746782

[bibr14-03010066211072466] Haddock C. K. Rindskopf D. Shadish W. R. (1998). Using odds ratios as effect sizes for meta-analysis of dichotomous data: A primer on methods and issues. Psychological Methods, 3(3), 339. 10.1037/1082-989X.3.3.339

[bibr15-03010066211072466] Haith A. M. Krakauer J. W. (2018). The multiple effects of practice: Skill, habit and reduced cognitive load. Current Opinion in Behavioral Sciences, 20, 196–201. 10.1016/j.cobeha.2018.01.015 30944847 PMC6443249

[bibr16-03010066211072466] * Ho A. M. H. Leung J. Y. Mizubuti G. B. Contardi L. H. Chan M. T. Lo T. S. Lee A. K. (2017). Inattentional blindness in anesthesiology: A simulation study. Journal of Clinical Anesthesia, 42, 36–39. 10.1016/j.jclinane.2017.07.015 28802148

[bibr17-03010066211072466] Hyman I. E. Boss S. M. Wise B. M. McKenzie K. E. Caggiano J. M. (2009). Did you see the unicycling clown? Inattentional blindness while walking and talking on a cell phone. Applied Cognitive Psychology, 24(5), 597–607. 10.1002/acp.1638

[bibr18-03010066211072466] Jensen M. S. Yao R. Street W. N. Simons D. J. (2011). Change blindness and inattentional blindness. WIREs Cognitive Science, 2(5), 529–546. 10.1002/wcs.130 26302304

[bibr19-03010066211072466] Mack A. Rock I. (1998). Inattentional blindness. MIT Press.

[bibr20-03010066211072466] McShane B. B. Böckenholt U. Hansen K. T. (2016). Adjusting for publication bias in meta-analysis: An evaluation of selection methods and some cautionary notes. Perspectives on Psychological Science, 11(5), 730–749. 10.1177/1745691616662243 27694467

[bibr21-03010066211072466] * Memmert D. (2006). The effects of eye movements, age, and expertise on inattentional blindness. Consciousness and Cognition, 15(3), 620–627. 10.1016/j.concog.2006.01.001 16487725

[bibr22-03010066211072466] Memmert D. Simons D. J. Grimme T. (2009). The relationship between visual attention and expertise in sports. Psychology of Sport and Exercise, 10(1), 146–151. 10.1016/j.psychsport.2008.06.002

[bibr23-03010066211072466] Most S. B. Scholl B. J. Clifford E. R. Simons D. J. (2005). What you see is what you set: Sustained inattentional blindness and the capture of awareness. Psychological Review, 112(1), 217–242. 10.1037/0033-295X.112.1.217 15631594

[bibr24-03010066211072466] Most S. B. Simons D. J. Scholl B. J. Jimenez R. Clifford E. Chabris C. F. (2001). How not to be seen: The contribution of similarity and selective ignoring to sustained inattentional blindness. Psychological Science, 12(1), 9–17. 10.1111/1467-9280.00303 11294235

[bibr25-03010066211072466] Murphy G. Greene C. M. (2016). Perceptual load induces inattentional blindness in drivers. Applied Cognitive Psychology, 30(3), 479–483. 10.1002/acp.3216

[bibr26-03010066211072466] * Näsholm E. Rohlfing S. Sauer J. D. (2014). Pirate stealth or inattentional blindness? The effects of target relevance and sustained attention on security monitoring for experienced and naïve operators. PLoS One, *9*:e86157. 10.1371/journal.pone.0086157 PMC3897661 24465932

[bibr27-03010066211072466] Neisser U. Becklen R. (1975). Selective looking: Attending to visually specified events. Cognitive Psychology, 7(4), 480–494. 10.1016/0010-0285(75)90019-5

[bibr28-03010066211072466] Pammer K. Blink C. (2018). Visual processing in expert drivers: What makes expert drivers expert? Transportation Research Part F: Traffic Psychology and Behaviour, 55, 353–364. 10.1016/j.trf.2018.03.009

[bibr29-03010066211072466] * Pammer K. Raineri A. Beanland V. Bell J. Borzycki M. (2018). Expert drivers are better than non-expert drivers at rejecting unimportant information in static driving scenes. Transportation Research Part F: Traffic Psychology and Behaviour, 59, 389–400. 10.1016/j.trf.2018.09.020

[bibr30-03010066211072466] Pashler H. (1994). Dual-task interference in simple tasks: Data and theory. Psychological Bulletin, 116(2), 220–244. 10.1037/0033-2909.116.2.220 7972591

[bibr31-03010066211072466] R Core Team (2019). R: A language and environment for statistical computing. R Foundation for Statistical Computing, Vienna, Austria. https://www.R-project.org/

[bibr32-03010066211072466] * Sannes A. C. Chaibi A. McCarthy P. W. (2018). More than meets the eye: Inattentional blindness. International Journal of Radiology and Imaging Technology, 4(2), 1–5. 10.23937/2572-3235.1510037

[bibr33-03010066211072466] Simons D. J. (2010). Monkeying around with the gorillas in our midst: Familiarity with an inattentional-blindness task does not improve the detection of unexpected events. I-Perception, 1(1), 3–6. 10.1068/i0386 23397479 PMC3563049

[bibr34-03010066211072466] Simons D. J. Chabris C. F. (1999). Gorillas in our midst: Sustained inattentional blindness for dynamic events. Perception, 28(9), 1059–1074. 10.1068/p281059 10694957

[bibr35-03010066211072466] Simons D. J. Jensen M. S. (2009). The effects of individual differences and task difficulty on inattentional blindness. Psychonomic Bulletin & Review, 16(6), 398–403. 10.3758/PBR.16.2.398 19293113

[bibr36-03010066211072466] * Simons D. J. Schlosser M. D. (2017). Inattentional blindness for a gun during a simulated police vehicle stop. Cognitive Research: Principles and Implications, 2, 1–8. 10.1186/s41235-017-0074-3 28989954 PMC5605606

[bibr37-03010066211072466] Viechtbauer W. (2010). Conducting meta-analyses in R with the metafor package. Journal of Statistical Software, 36(3), 1–48. 10.18637/jss.v036.i03

