# Supplementary figures and images for: Does Expertise Reduce Rates of Inattentional Blindness? A Meta-Analysis
Source: Perception. 2022 Jan 21;51(2):131–47. doi: 10.1177/03010066211072466 (PMC8813586; doi:10.1177/03010066211072466)

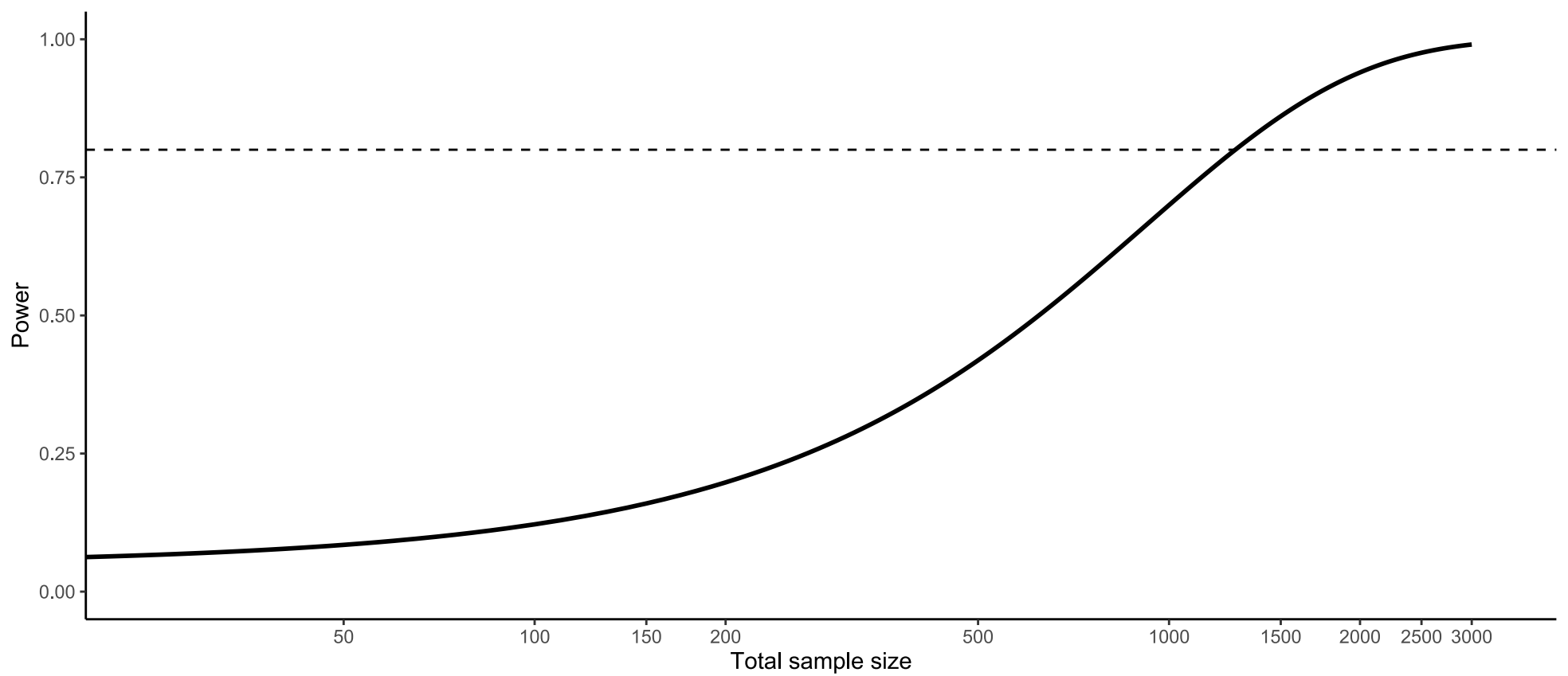

Power curve for an Odds Ratio = 1.33

Supplement: sj-pdf-1-pec-10.1177_03010066211072466 - Supplemental material for Does Expertise Reduce Rates of Inattentional Blindness? A Meta-Analysis [file sj-pdf-1-pec-10.1177_03010066211072466.pdf]
